# Do labeled versus unlabeled treatments of alternatives’ names influence stated choice outputs? Results from a mode choice study

**DOI:** 10.1371/journal.pone.0178826

**Published:** 2017-08-14

**Authors:** Wen Jin, Hai Jiang, Yimin Liu, Erica Klampfl

**Affiliations:** 1 Department of Industrial Engineering, Tsinghua University, Beijing 100084, China; 2 Research & Advanced Engineering, Ford Motor Company, 2101 Village Road MD-2149, Dearborn, MI 48121, United States of America; Beihang University, CHINA

## Abstract

Discrete choice experiments have been widely applied to elicit behavioral preferences in the literature. In many of these experiments, the alternatives are *named alternatives*, meaning that they are naturally associated with specific names. For example, in a mode choice study, the alternatives can be associated with names such as car, taxi, bus, and subway. A fundamental issue that arises in stated choice experiments is whether to treat the alternatives’ names as labels (that is, labeled treatment), or as attributes (that is, unlabeled treatment) in the design as well as the presentation phases of the choice sets. In this research, we investigate the impact of labeled versus unlabeled treatments of alternatives’ names on the outcome of stated choice experiments, a question that has not been thoroughly investigated in the literature. Using results from a mode choice study, we find that the labeled or the unlabeled treatment of alternatives’ names in either the design or the presentation phase of the choice experiment does not statistically affect the estimates of the coefficient parameters. We then proceed to measure the influence toward the willingness-to-pay (WTP) estimates. By using a random-effects model to relate the conditional WTP estimates to the socioeconomic characteristics of the individuals and the labeled versus unlabeled treatments of alternatives’ names, we find that: a) Given the treatment of alternatives’ names in the presentation phase, the treatment of alternatives’ names in the design phase does not statistically affect the estimates of the WTP measures; and b) Given the treatment of alternatives’ names in the design phase, the labeled treatment of alternatives’ names in the presentation phase causes the corresponding WTP estimates to be slightly higher.

## Introduction

Discrete choice experiments have been widely applied to elicit behavioral preferences in many fields such as transportation [1–3], marketing [4], environmental research [5, 6], health economics [7, 8], and housing studies [9, 10]. In these experiments, respondents are asked to complete one or more choice tasks, each of which contains several alternatives with hypothetical attribute values. Within each choice task, the respondent needs to indicate his or her most preferred alternative, which can later be used to estimate appropriate discrete choice models and quantify respondents’ preferences toward the attributes. In many of these experiments, the alternatives are *named alternatives*, meaning that they are naturally associated with specific names, which convey additional information regarding the tangible or intangible qualities of the alternatives [11]. For example, in mode choice studies, the alternatives’ names can be car, taxi, bus, and subway [12, 13]; in recreational site choice, the names can be river walk, hill walk, and field walk [14]; in air travel choice, the names can be Air France, KLM, and Iberia [15, 16]; and in the choice of colorectal cancer screening methods, the names can be fecal occult blood tests (FOBTs), sigmoidoscopy, and colonoscopy [17].

A fundamental issue that arises in stated choice experiments involving named alternatives is whether to treat the alternatives’ names as labels (that is, labeled treatment), or as attributes (that is, unlabeled treatment). These decisions have important ramifications toward the design and the presentation of the choice sets:

- In the design of the choice sets, that is, the process that generates the underlying variations of the attribute values to maximize identification capabilities of discrete choice models, the labeled treatment of alternatives’ names views the names as titles of the alternatives, while the unlabeled treatment view the names as attributes of the alternatives. The labeled or unlabeled treatment of alternatives’ names necessitates either the labeled or the unlabeled design method [18]. The labeled design method can easily generate realistic choice tasks because it accommodates alternative specific attributes and attribute levels. Since we must allocate one alternative in the choice set for each distinct alternative name, the number of alternatives in the choice sets can become rather large when we have four or more distinct alternatives’ names, which may place considerable cognitive burden on the respondents [19]. The unlabeled design methods, however, treat the alternatives’ names as attributes. Therefore, it offers more flexibility with respect to the number of alternatives to include in a choice set. In addition, by restricting certain combinations of attribute levels, it can also avoid generating unrealistic combinations of attribute levels;
- In the presentation of the choice sets, that is, the process when we show the choice sets to the respondents and solicit their responses, the labeled treatment displays the alternatives’ names as their labels in the header row of the choice task as is illustrated by the example shown in Table 1. We refer to this as the labeled presentation style and since the alternatives’ names are displayed rather prominently, it more or less affects how respondents reach their choice outcomes [18]. The unlabeled treatment presents the alternatives’ names as attributes while referencing the alternatives with generic names, such as “Option 1”, “Option 2”, and “Option 3”, in the header row as is shown by the example in Table 2. Since the unlabeled treatment displays the alternatives’ names along with the other attributes, it could potentially force the respondents to compare the attribute values before reaching their decisions.

**Table 1:** An example for the labeled treatment of alternatives’ names in the presentation phase. The names (that is, car, bus, and subway) are displayed in the header row of the choice task.

	Car	Bus	Subway
Travel time	20 min.	30 min.	15 min.
Travel cost	20 Yuan	0.4 Yuan	2 Yuan
Your choice	□	□	□

**Table 2:** An example for the unlabeled treatment of alternatives’ names in the presentation phase. The names (that is, car, bus, and subway) are displayed in the row that corresponds to “travel mode”.

	Option 1	Option 2	Option 3
Travel time	20 min.	30 min.	15 min.
Travel cost	20 Yuan	0.4 Yuan	2 Yuan
Travel mode	Car	Bus	Subway
Your choice	□	□	□

**Fig 1:**
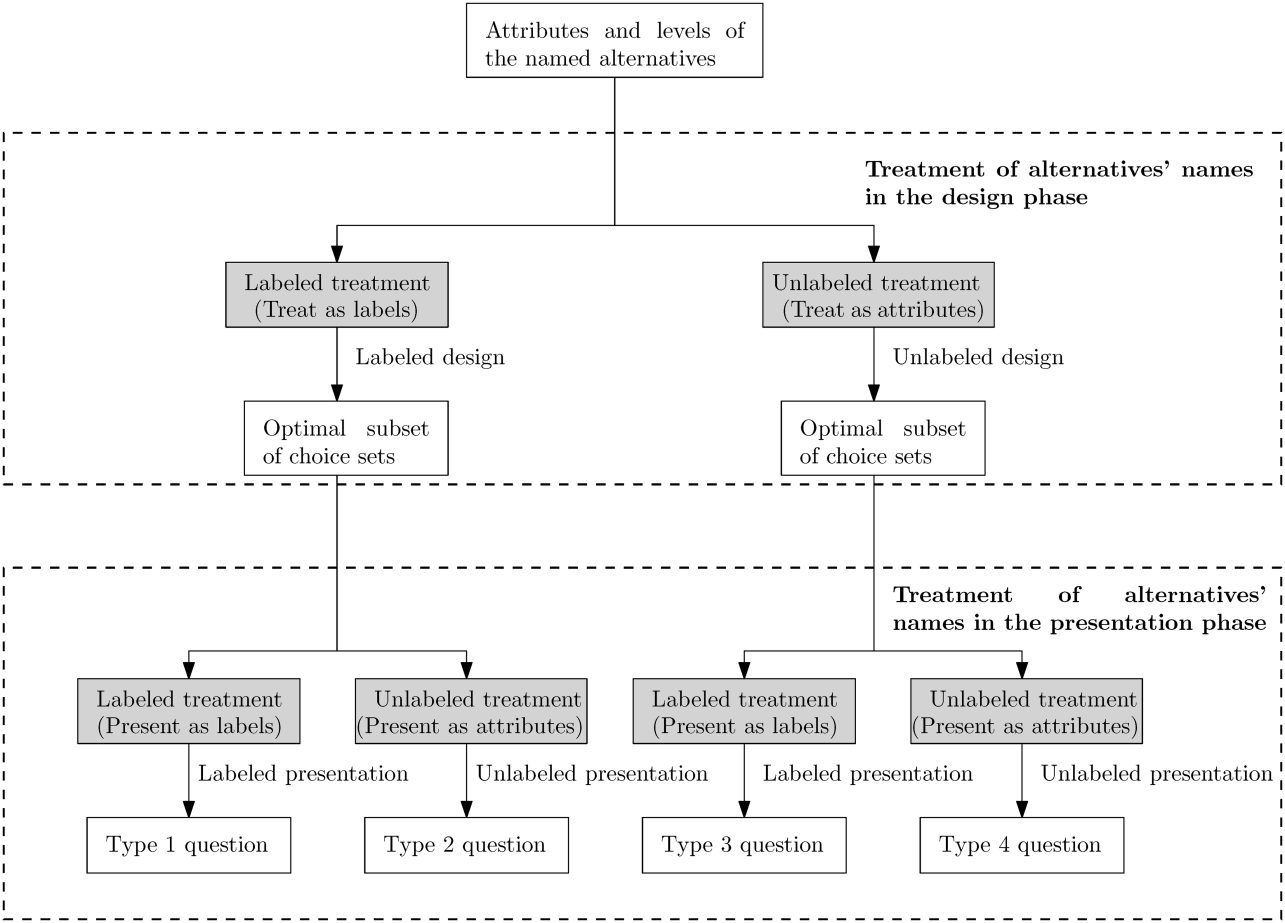
The labeled versus unlabeled treatments of alternatives’ names in the design and the presentation phases produce four types of questions for a choice experiment.

The major contribution of this research is that we investigate the impact of labeled versus unlabeled treatments of alternatives’ names on the outcome of stated choice experiments involving named alternatives, a problem that has not been thoroughly investigated in the literature. Since the decision to use either the labeled or unlabeled treatment of alternatives’ names in the design phase is independent of that in the presentation phase, for a stated choice experiment involving named alternatives we can produce four types of questions as are shown in Fig 1. In the very beginning of a choice experiment, we need to determine the set of alternatives, their attributes, and the associated attribute levels. Then, depending on the treatment of alternatives’ names in the design phase, we adopt either the labeled or the unlabeled design method to obtain the corresponding optimal subset of choice sets. Regardless of which treatment we take in the design phase, when presenting the choice sets in the optimal subset to the respondent, we can choose the labeled or unlabeled treatment of alternatives’ names in the presentation phase. Note that in the literature, there is also a fifth type of question, where the alternatives’ names are completely removed and the cognitive significance of the alternatives’ names are examined. The fifth type of questions essentially turns the named alternatives into *non-named alternatives*, and the unlabeled design method and the unlabeled presentation style have to be adopted because the alternatives’ names are no longer there. Table 3 shows the relationship among the five types of questions. The rows and the columns indicate the three treatments of alternatives’ names in the design and presentation phases, respectively.

**Table 3:** If we allow the alternatives’ names to be removed, altogether five types of questions involving named alternatives can be produced.

		**Treatment of names in the presentation phase**		
		Labeled treatment (Present as labels)	Unlabeled treatment (Present as attributes)	Remove the names
**Treatment of names in the design phase**	Labeled treatment (Treat as labels)	**Type 1 question** [20] [17] [6] [14] This paper	**Type 2 question** This paper	—
	Unlabeled treatment (Treat as attributes)	**Type 3 question** This paper	**Type 4 question** This paper	—
	Remove the names	—	—	**Type 5 question** [20] [17] [6]

Existing literature investigating the impact of labeled versus unlabeled treatments of alternatives’ names in stated choice experiments is scarce. Table 3 summarizes the types of questions compared in previous research and we notice that most, if not all, research focuses on comparing Type 1 and Type 5 questions. In an effort to understand the influence of policy labels in environmental choice modeling, [20] study the effects of employing labeled rather than generic choice set configurations. In their work, Types 1 and 5 questions are generated and given to a split sample. Results show that while the mean unobserved utility component differs, the vectors of attribute taste parameters do not differ significantly when scale differences are accommodated. Later, [17] compare the feasibility, dominant effects, and convergent validity between Types 1 and 5 questions in the context of colorectal cancer screening programs in the Netherlands. They find that the inclusion of labels plays a significant role in individual choices and reduces the attention to the actual attributes. [6] assess the effect of energy technology labels on preferences. They find that the use of labeled alternatives leads to significantly different estimated parameters, while most implicit prices and welfare measures are found to be statistically equivalent. [14] investigate whether and to what extent respondents reach their choices based on the labels only. They employ a discrete mixture modeling approach on data collected using Type 1 choice tasks. They find that a portion of respondents reach their choices based on the alternatives’ names only and show that this has a large impact toward welfare measures.

In this research, we compare the first four types of questions in Table 3 that include the alternatives’ names in both the design and the presentation of the choice sets. We quantify the influence associated with the labeled versus unlabeled treatments toward stated choice outputs in the context of a mode choice study, where residents’ preferences to public transportation, private car, taxi, and vehicle sharing are studied. We generate choice tasks for each type of questions in our survey and ask respondents to indicate their preferred travel mode for each choice task. We then estimate mixed logit models using responses to each question type to assess the influence of the different treatments toward parameter estimates. Finally, we analyze the conditional willingness-to-pay (WTP) measures estimated from these data sets using a random-effects model.

The remainder of this paper is organized as follows. Section 2 gives the background of named alternatives and discuss in detail their implications in stated choice experiments. Section 3 briefly reviews the mixed logit model, the likelihood ratio test for parameter comparison, the conditional WTP estimates, and the random-effects model used in our analysis. Section 4 introduces the mode choice study, the design and the administration of the survey. Section 5 presents the results of the case study and our findings. Finally, we conclude our discussion in Section 6.

## Named alternatives and stated choice experiments

In Section 2.1 we discuss the implications associated with the treatment of alternatives’ names in the design of the choice sets, and in Section 2.2 we discuss that associated with the treatment of alternatives’ names in the presentation of the choice sets.

### Treatment of alternative’s name in the design of the choice sets

In the design of the choice sets, we generate the underlying variations of the attribute values to maximize our model’s identification capabilities. The labeled treatment of alternatives’ names views the alternatives’ names as labels (or titles), while the unlabeled treatment views the names as attributes. Depending on the treatment, we need to employ either the labeled or the unlabeled design method [18]. In the labeled design method, the alternatives’ names are treated as labels and alternatives with different labels come from different candidate alternative sets. In the unlabeled design method, the names are treated as attributes and the alternatives all come from the same candidate alternative set.

The two design methods have their respective benefits and drawbacks. First of all, in the labeled design method, the number of alternatives in a choice set typically equals the number of distinct alternatives’ names. That is, one for each distinct alternative’s name. For example, in a mode choice study involving five different transportation modes, the choice sets generated by the labeled design method typically need to have exactly five alternatives. When the number of distinct alternatives’ names grows, the number of alternatives in the choice set also increases, which may creates information overload to the respondents and negatively impact their decision process. The unlabeled design method, however, gives us the flexibility with respect to the number of alternatives in the choice set, which can help reduce the choice complexity when the alternatives have a large number of distinct names.

Secondly, the universal choice set, that is, the set of all possible choice sets, in the labeled design is typically smaller than that in the unlabeled design, which makes the labeled design method computationally amenable when we search for the optimal design, that is, the optimal subset of choice sets. Suppose that the alternatives we consider have *M* distinct names and we have decided that the numbers of alternatives in the choice sets are *M* for both design methods. Let us further assume that in addition to its name, each of the alternatives has *A* attributes with *L* levels. Then, there are *L*^*MA*^ possible choice sets under the labeled design. In the unlabeled design, however, because the names are treated as attributes, we now have *ML*^*A*^ possible alternatives that can be included in each of the *M* positions of a choice set. Since identical alternatives are not allowed to appear in the same choice set, there are “*ML*^*A*^ choose *M*” possible choice sets under the unlabeled design. For a typical choice experiment with four alternatives, five attributes (excluding the names of the alternatives), and three levels, the numbers of possible choice sets in the labeled design is 3^(4×5)^ = 3, 486, 784, 401. However, in the unlabeled design, the number of possible alternatives is 4 × 3^5^ = 972 and the number of possible choice sets is $C_{972}^{4}=36,963,217,215$, which is substantially greater than that in the labeled design. Note that in the unlabeled design method, we can reduce the size of the universal choice set by decreasing the number of alternatives in the choice set. In the previous example, if we display three alternatives in each choice set, the number of possible choice sets can then be reduced to $C_{972}^{3}=152,582,940$.

Thirdly, the choice set produced by the labeled design method typically has one alternative for each distinct name, which may not be desirable when respondents make decisions solely based on the alternatives’ names. For example, in a mode choice study, some respondent may always choose to drive a car regardless of the actual attributes associated with the alternatives. This attribute non-attendance phenomenon has been widely recognized in the literature [14]. The unlabeled design method, however, treats the alternatives’ names as attributes, which means that in the choice set generated there can be more than one alternative having the same name. For example, there can be two car alternatives with different travel times and costs. This is advantageous because having two car alternatives can force the respondents to pay attention to the actual attribute values even if he wants the car alternative.

A review of the literature shows that there is no consensus with regard to which design method is more appropriate for stated choice experiments involving named alternatives. We find that the labeled design method is often used in transportation related studies [21–25], while the unlabeled design method is commonly adopted by health and marketing related studies [11, 17].

### Treatment of alternatives’ names in the presentation of the choice set

It has been recognized that the presentation of the choice tasks affects choice outcomes, for example, the order of the alternatives, the order of the attributes, the appearance of the choice tasks, and so on [26, 27]. For choice experiments involving named alternatives, regardless of the type of experimental design method used to generate the choice set, we have two ways to present them depending on where we display the alternatives’ names: a) the labeled presentation style, where the names are presented in the header row of the table as is illustrated by the example shown in Table 1. In this example, the alternatives’ names, that is, car, bus, and subway, are displayed in the header row as if they are the labels of the alternatives; and b) the unlabeled presentation style, where the names are presented together with the other attributes as is illustrated by the example shown in Table 2. In this example, the alternatives’ names are displayed along with travel times and travel costs.

A potential issue with the labeled presentation style is that respondents may base their choices wholly or largely on the prominently displayed name and pay less attention to the actual attributes associated with the alternatives. In the literature, there has been widespread evidence that respondents often use heuristics when making their choices [17, 28, 29]. [30], [31], and [32] investigate the extent to which respondents employ information processing strategies in their decision process. [14] find evidence that a proportion of respondents reach their choices solely on the basis of the alternative’s label.

For ease of exposition in the following text, we sometimes use the phrase “labeled (or unlabeled) design” as a shorthand for the labeled (or unlabeled) treatment of alternatives’ names in the design phase, and the phrase “labeled (or unlabeled) presentation” as a shorthand for the labeled (or unlabeled) treatment of alternatives’ names in the presentation phase.

## Methods

In this research, we are interested in quantifying the impact of labeled versus unlabeled treatments of alternatives’ names on both the parameter estimates and the WTP measures in stated choice experiments. Since there are two kinds of treatments available for the alternatives’ names in both the design and the presentation phases of the choice experiment, we can produce four types of questions shown in Fig 1, and use them to solicit responses from the respondents. These responses form four data sets based on the types of questions used. The parameter estimates and WTP measures obtained from these data sets are then compared and analyzed using methods detailed in this section. We first present in Section 3.1 a brief review of the mixed logit model, which is widely used in the field of tranportation studies [33, 34]. We then discuss the statistical tests to compare parameter estimates across data sets in Section 3.2. Finally, in Sections 3.3 and 3.4 we show how conditional WTP estimates can be obtained from the mixed logit model and how the random-effects model can be used to explain the variation in WTP by the socioeconomic characteristics of the individuals as well as the different treatments of alternatives’ names.

### The mixed logit model

Let us assume that we have *N* individuals indexed by *n* and each of them faces *T* types of questions indexed by *t*. Given question type *t*, there are *S* choice tasks indexed by *s*. In the following text, we briefly review the mixed logit model and its estimation based on responses collected for Type *t* questions. Let *C*_*stn*_ be the set of alternatives in the *s*-th choice task of question type *t* faced by individual *n*. Let *y*_*istn*_ take value 1 if respondent *n* chooses alternative *i* in *C*_*stn*_; and 0, otherwise. Define ***y***_*stn*_ = (*y*_1*stn*_, *y*_2*stn*_, ⋯, *y*_|*C*_*stn*_|*stn*_), where |*C*_*stn*_| is the cardinality of set *C*_*stn*_. Let ***y***_*tn*_ = (***y***_1*tn*_, *y*_2*tn*_, ⋯, ***y***_*Stn*_) be the observed choices from individual *n* for all choice tasks of type *t* and ***Y***_*t*_ = (***y***_*t*1_, ***y***_*t*2_, ⋯, ***y***_*tN*_) be the observed choices from all individuals for all choice tasks of type *t*. Let ***β***_*tn*_ be the coefficient vector for individual *n* when facing question type *t*. The utility function for alternative *i* ∈ *C*_*stn*_ can be written as

$$\begin{array}{c} U_{istn}=\beta_{tn}^{\prime }x_{istn}+\epsilon_{istn}, \end{array}$$

where ***x***_*istn*_ is a vector of length *κ*, which corresponds to the observed attributes related to alternative *i* in choice task *s* for type *t* questions faced by individual *n*; and *ϵ*_*istn*_ is the random term representing the unobserved utility component. In the mixed logit model, ***β***_*tn*_ is assumed to vary over individuals to capture preference heterogeneity in the population. It is common to assume that ***β***_*tn*_ conforms to a multivariate normal distribution with mean ***b***_*t*_ and variance-covariance matrix ***W***_*t*_, and its density function is written as *ϕ*(***β***_*tn*_|***b***_*t*_, ***W***_*t*_). The probability for individual *n* to choose alternative *i* in choice set *C*_*stn*_ conditional on ***β***_*tn*_ is defined as

$$\begin{array}{c} P_{stn}\left(i|\beta_{tn},C_{stn}\right)=\frac{\text{exp}(\beta_{tn}^{{}^{\prime }}x_{istn})}{\sum_{j\in C_{stn}}\text{exp}\left(\beta_{tn}^{{}^{\prime }}x_{jstn}\right)}. \end{array}$$

The probability of individual *n*’s choices for all *S* choice tasks conditional on ***β***_*tn*_ is

$$\begin{array}{c} L(y_{tn}|\beta_{tn})=\prod_{s=1}^{S}\prod_{i\in C_{stn}}{\left(\frac{\text{exp}\left(\beta_{tn}^{{}^{\prime }}x_{istn}\right)}{\sum_{j\in C_{stn}}\text{exp}\left(\beta_{tn}^{{}^{\prime }}x_{jstn}\right)}\right)}^{y_{istn}}. \end{array}$$

The unconditional probability is therefore the integral of *L*(***y***_*tn*_|***β***_*tn*_) over ***β***_*tn*_

$$\begin{array}{c} L\left(y_{tn}|b_{t},W_{t}\right)=\int L\left(y_{tn}|\beta_{tn}\right)\phi \left(\beta_{tn}|b_{t},W_{t}\right)d\beta_{tn}, \end{array}$$

and the final likelihood function is

$$\begin{array}{c} L\left(b_{t},W_{t}\right)=\prod_{n=1}^{N}L\left(y_{tn}|b_{t},W_{t}\right). \end{array}$$

By maximizing the above likelihood function, we can obtain the estimates for ***b***_*t*_ and ***W***_*t*_. In this research, we estimate parameters ***b***_*t*_ and ***W***_*t*_ using the hierarchical Bayes approach based on the Markov Chain Monte Carlo method, which offers two advantages over the maximum simulated likelihood approach: a) It does not attempt to maximize the likelihood function, which is very challenging numerically and convergence can be hard to achieve; and b) Desirable estimation properties, such as consistency and efficiency, can be obtained under more relaxed conditions [33]. Let *k*(***b***_*t*_, ***W***_*t*_) be the prior density function for ***b***_*t*_ and ***W***_*t*_, then the posterior distribution of ***b***_*t*_ and ***W***_*t*_ is

$$\begin{array}{c} K\left(b_{t},W_{t}|Y_{t}\right)\propto \prod_{n=1}^{N}L\left(y_{tn}|b_{t},W_{t}\right)k\left(b_{t},W_{t}\right). \end{array}$$

It is customary to assume that ***b***_*t*_ and ***W***_*t*_ are independent, the prior on ***b***_*t*_ is normal with an unboundedly large variance, and the prior on ***W***_*t*_ is inverted Wishart with *κ* degrees of freedom and scale matrix ***I***, which is an identity matrix with rank *κ*. To improve computational efficiency, the hierarchical Bayes approach treats each ***β***_*tn*_ as a parameter along with ***b***_*t*_ and ***W***_*t*_, and the corresponding joint posterior for ***b***_*t*_, ***W***_*t*_, and ***β***_*tn*_ ∀*n* is then given by

$$\begin{array}{c} K\left(b_{t},W_{t},\beta_{tn}\;\forall n|Y_{t}\right)\propto \prod_{n=1}^{N}L\left(y_{tn}|\beta_{tn}\right)\phi \left(\beta_{tn}|b_{t},W_{t}\right)k\left(b_{t},W_{t}\right), \end{array}$$ (1)

which can be efficiently drawn using the Gibbs sampler [33].

### Likelihood ratio test for parameter comparison

Note that there are two types of parameters in the mixed logit model: a) the coefficient parameters, that is, ***b***_*t*_ and ***W***_*t*_, which describe ***β***_*tn*_, the coefficients for the attributes; and b) the scale parameter, which measures the variability of the random term *ϵ*_*istn*_. When estimating the mixed logit model based on a single data set, the scale parameter is often normalized to one [33]. However, when several mixed logit models with the same specification are estimated on different data sets, it is then necessary to control for the differences in the scale parameters. This is typically done by normalizing the scale parameter associated with one of the data sets to one and estimating the scale parameters associated with the others.

Let *D*_1_, *D*_2_, ⋯, *D*_*T*_ be the *T* data sets we collect (that is, one data set for each of the *T* types of questions), we can estimate *T* mixed logit models and get the corresponding parameter estimates. We want to see whether the parameters, that is, the coefficient parameters and the scale parameters, are the same across these data sets. This can be accomplished by the likelihood ratio test, whose steps for the mixed logit model are similar to those for the multinomial logit model and we just need to account for the potential difference in the scale parameters of the data sets [35].

Suppose that we want to compare the parameter estimates obtained from Type 1 and Type 2 questions. For data set *D*_*t*_, where *t* ∈ {1, 2}, let ***b***_*t*_ and ***W***_*t*_ be the coefficient parameters and *μ*_*t*_ be corresponding scale parameter of the mixed logit model. The likelihood ratio test proceeds in two steps. In the first step, we test the hypothesis that the coefficient parameters are identical while allowing the scale parameters to be different. That is,

$$\begin{array}{c} H_{a}:\;b_{1}=b_{2}\;\text{and}\;W_{1}=W_{2}. \end{array}$$ (2)

The likelihood ratio test involves estimating an unrestricted model and a restricted one. The unrestricted model consists of two separate mixed logit models, where the first one is fit over data set *D*_1_ and the second one is fit over data set *D*_2_, independently of each other. The unrestricted model essentially returns the estimates for ***b***_1_, ***b***_2_, ***W***_1_, and ***W***_2_. In addition, the construction of the unrestricted model implies that we allow the scale parameters in *D*_1_ and *D*_2_ to be different. Let the sum of the loglikelihood function values for the two mixed logit models be *L*_1_, which is also referred to as the loglikelihood for the unrestricted model. The restricted model is obtained by enforcing *H*_*a*_ in the unrestricted model. This can be conveniently achieved by estimating a mixed logit model over the following data matrix

$$\begin{array}{c} \Omega =[\begin{array}{c} D_{1}\\ \mu_{2}D_{2} \end{array}], \end{array}$$

which is created by multiplying all independent variables in *D*_2_ by the scale parameter *μ*_2_, whose value let us for now assume is known, and then concatenating with *D*_1_. This implies that we normalize *μ*_1_, the scale parameter for *D*_1_, to 1, and that *μ*_2_ now reflects the relative scale of *D*_2_ with respect to *D*_1_. The above construction imposes the restriction that ***b***_1_ = ***b***_2_ and ***W***_1_ = ***W***_2_ while allowing the scale parameters to be different. The estimation of the mixed logit model for Ω is conducted by systematically varying the value of *μ*_2_ and finding the one that maximizes the value of the maximum loglikelihood function. Let the maximum value be *L*_2_. The test statistic for *H*_*a*_ is −2(*L*_2_ − *L*_1_), which is *χ*^2^ distributed with 2*κ* − 1 degrees of freedom.

If *H*_*a*_ cannot be rejected, we proceed to the second step and test the hypothesis that the scale parameters are identical between *D*_1_ and *D*_2_, that is,

$$\begin{array}{c} H_{b}:\;\mu_{1}=\mu_{2}. \end{array}$$ (3)

This can be achieved by conducting a second likelihood ratio test, where the unrestricted model is estimated based on data matrix Ω and the restricted model is estimated based on data matrix Ω′ defined below

$$\begin{array}{c} \Omega^{\prime }=[\begin{array}{c} D_{1}\\ D_{2} \end{array}]. \end{array}$$ (4)

This implies that we equalize *μ*_1_ and *μ*_2_, and that both are normalized to one. Let *L*_3_ be the loglikelihood for the model based on Ω′ and the test statistics is −2(*L*_3_ − *L*_2_), which is *χ*^2^ distributed with 1 degree of freedom. The above procedure can be easily extended to situations where we need to compare parameter estimates across more than two data sets as is shown in Section 5.1.

### Conditional WTP estimates

A key output of a discrete choice model is the WTP measurement for various attributes of the alternatives. In addition to producing the WTP estimates for the entire sample, the mixed logit model can also estimate each individual’s WTP conditional upon his or her observed choices. We can then conduct further analysis, for example, using a random-effects model, to relate the variation in the individual specific WTP to factors such as the characteristics of the individuals and the treatment of alternatives’ names. This type of two-step analysis, that is, a mixed logit model followed by a random-effects model, is first proposed by [5] to estimate the economic benefits associated with rural landscape improvement. Recently, [9] and [36] apply this method to analyze preference heterogeneity in residential choice problems.

Recall that when estimating the mixed logit model based on ***Y***_*t*_ in Section 3.1, that is, the responses to type *t* questions, through the hierarchical Bayes method, each individual’s ***β***_*tn*_ is treated as a random variable along with ***b***_*t*_ and ***W***_*t*_. Based on the joint posterior for ***b***_*t*_, ***W***_*t*_, and ***β***_*tn*_ ∀*n* defined in Eq (1), we can obtain the posterior distribution of ***β***_*tn*_ by integrating out variables ***b***_*t*_, ***W***_*t*_, ***β***_*t*1_, ***β***_*t*2_, ⋯, ***β***_*t*, *n*−1_, ***β***_*t*, *n*+1_, ⋯, ***β***_*tN*_ in the joint posterior:

$$\begin{array}{c} h(\beta_{tn}|Y_{t})=\int \int \cdots \int K\left(b_{t},W_{t},\beta_{tn}\forall n|Y_{t}\right)\;\text{d}b_{t}\;\text{d}W_{t}\;\text{d}\beta_{t1}\;\text{d}\beta_{t2}\cdots \;\text{d}\beta_{t,n-1}\;\text{d}\beta_{t,n+1}\cdots \;\text{d}\beta_{tN}. \end{array}$$ (5)

As a result, the mean ***β***_*tn*_ for individual *n* in the sampled population can be written as

$$\begin{array}{c} {\bar{\beta }}_{tn}=E[\beta_{tn}|Y_{t}]=\int \beta_{tn}h\left(\beta_{tn}|Y_{t}\right)\;\text{d}\beta_{tn} \end{array}$$ (6)

$$\begin{array}{cc} & =\int \int \cdots \int \beta_{tn}K\left(b_{t},W_{t},\beta_{tn}\forall n|Y_{t}\right)\;\text{d}b_{t}\;\text{d}W_{t}\;\text{d}\beta_{t1}\;\text{d}\beta_{t2}\cdots \;\text{d}\beta_{tN} \end{array}$$ (7)

The last equality in Eq (7) is obtained by plugging in the result for *h*(***β***_*tn*_|***Y***_*t*_) from Eq (5). The above multi-dimensional integral does not have a closed form and is calculated by simulation. Note that in the hierarchical Bayes approach, we have already obtained the draws for ***b***_*t*_, ***W***_*t*_, and ***β***_*tn*_ ∀*n* from their joint posterior defined in Eq (1). Let *R* be the number of draws after the burn-in period, and $\beta_{tn}^{r}$ be the sampled value in the *r*-th draw, we have

$$\begin{array}{c} {\bar{\beta }}_{tn}=\frac{1}{R}\sum_{r=1}^{R}\beta_{tn}^{r}. \end{array}$$ (8)

Let ${\bar{\beta }}_{tnj}$ be the coefficient for attribute *j* in vector ${\bar{\beta }}_{tn}$, then individual *n*’s WTP for attribute *j* is given by

$$\begin{array}{c} WTP_{tnj}=-\frac{{\bar{\beta }}_{tnj}}{\beta_{tp}}, \end{array}$$ (9)

where *β*_*tp*_ is the price coefficient. This individual level WTP estimate can then be related to the socioeconomic characteristics of each individual as well as the treatments of alternatives’ names.

### The random-effects model

Once we obtain the conditional WTP estimates for each individual and each question type, we can pool them together to create a panel. According to [37], panel data can be analyzed by three types of models: the pooled regression model, the fixed-effects model, and the random-effects model. We adopt the random-effects model because it is capable of controlling for unobserved heterogeneity among the individuals. Besides, it also allows us to include panel-invariant variables, for example, the socio-demographic characteristics of the individuals, among the regressors. The econometric specification of the random-effects model is as follows:

$$\begin{array}{c} y_{tn}=\beta^{\prime }x_{tn}+\left(\alpha +\nu_{n}\right)+\omega_{tn}, \end{array}$$

where *n* represents a given individual, *t* refers to a specific question type, ***y***_*tn*_ is the WTP estimate of individual *n* for question type *t*, ***x***_*tn*_ is the vector of explanatory variables, *α* is the intercept of the model, *ν*_*n*_ is the unobserved individual-specific error term, and *ω*_*tn*_ is the random disturbance. Note that the random-effects model divide the unobserved effect into an error term that is individual specific (*ν*_*n*_) and a component that is common to all individuals and question types (*ω*_*tn*_). The individual specific disturbance is constant across all WTP estimates observed for this individual regardless of the question type. Due to this treatment, our model is also referred to as the one-way random-effects model.

In our random-effects model, the dependent variable is the conditional WTP estimate specific to each individual and each question type. The independent regressors include the socioeconomic characteristics and life styles of the individuals, dummy variables indicating the labeled or unlabeled treatment of alternatives’ names in the design and the presentation phases of the choice experiment, and variables that describe the choice consistency of the individuals.

## Case study

The main objective of this research is to investigate the influence of labeled versus unlabeled treatments of alternatives’ names in stated choice experiments. We examine the problem in the context of a mode choice study involving car, taxi, public transportation, and vehicle sharing in the city of Beijing. Section 4.1 gives the background of the study. Sections 4.2 and 4.3 present the design and administration of the questionnaire, respectively.

### Background to the mode choice study

Beijing has been undergoing a rapid motorization process in the past two decades. This rapid motorization process has created significant economic benefits as well as severe congestion in the city. To alleviate congestion, Beijing has been exploring a variety of means [38, 39], among which vehicle sharing has received great attention [40]. Vehicle sharing, also known as car sharing, is a program through which individuals can rent vehicles for a short period of time, often by the hour. Unlike classical car rental, vehicles used in vehicle sharing are positioned in unstaffed neighbourhood locations. Since vehicle sharing turns the fixed costs of ownership into variable costs, it provides individuals and families with periodic access to automobiles with relatively low cost. The major social benefit of vehicle sharing is reduced vehicle ownership and use intensity [41, 42].

The municipal government in Beijing is very interested in knowing residents’ choice behavior between existing travel modes and vehicle sharing should it be introduced. This would help the government estimate its impact to vehicle ownership and/or usage. Moreover, the government also wants to identify the socioeconomic characteristics of the subpopulation that would favor vehicle sharing. This information can be used to better target potential vehicle sharing customers. However, little research has been conducted on Chinese residents’ preferences toward vehicle sharing. [43] carry out a survey within Beijing in spring 2006 to explore vehicle sharing familiarity and response. They report that over 25% of respondents expressed a high level of interest in vehicle sharing and those interested in vehicle sharing were skewed towards younger age categories, higher education levels, and slightly higher income. [44] study vehicle sharing in Shanghai and they find that those interested in vehicle sharing were more likely to be educated and had longer commuting times.

### Survey design

There are three major urban transportation modes in Beijing, that is, private car, taxi, and public transportation [45]. These three modes together with vehicle sharing form the four travel alternatives available to the respondents. These alternatives are named alternatives and the travel modes can be viewed as their names. Table 4 summarizes the attributes and their levels associated with these alternatives in our survey. The first row indicates the treatment of alternatives’ names: either as attributes, or as labels. The first column shows the attributes for the alternatives, that is, mode, travel cost, parking cost, in-vehicle time, out-of-vehicle time, and number of transfers. Note that we break down the overall cost into two components: 1) travel cost, which refers to the gasoline expense for private car, the fare for public transportation and taxi, or the rental cost for vehicle sharing; and 2) parking cost, which refers to the cost paid to park the private car or rental car at the destination. The second column shows the attribute levels in the unlabeled design when alternative’s names are treated as attributes. To prevent generating unrealistic alternatives, restrictions are applied to prohibit certain combinations of attribute levels in the unlabeled design. For example, when the mode is public transportation, the parking cost is restricted to zero. The third column shows the attribute levels in the labeled design when alternatives’ names are treated as labels. In this design, each alternative (or travel mode) has its own attribute levels.

**Table 4:** The attributes and their levels in our mode choice study.

Treatment of alternatives’ names in the design phase	Unlabeled treatment (Treat as attributes)	Labeled treatment (Treat as labels)	
Mode	Public Transportation (PT), Private car (PC) Taxi (TX) Vehicle sharing (VS)		
Travel cost (Yuan)	0.4, 2, 5, 15, 30, 45	Public transportation:	0.4, 2, 5
		Private car:	15, 30, 45
		Taxi:	15, 30, 45
		Vehicle sharing:	15, 30, 45
Parking cost (Yuan)	0, 10, 20	Public transportation:	0
		Private car:	0, 10, 20
		Taxi:	0
		Vehicle sharing:	0, 10, 20
In-vehicle time (min)	20, 40, 60	Public transportation:	40, 50, 60
		Private car:	20, 30, 40
		Taxi:	20, 30, 40
		Vehicle sharing:	20, 30, 40
Out-of-vehicle time (min)	5, 10, 15, 20	Public transportation:	10, 15, 20
		Private car:	5, 10, 15
		Taxi:	10, 15, 20
		Vehicle sharing:	5, 10, 15
Number of transfers	0, 1, 2	Public transportation:	0, 1, 2
		Private car:	0
		Taxi:	0
		Vehicle sharing:	0

Given the attributes and their levels in Table 4, we use the D-optimal design SAS macros provided by [46] to come up with the optimal subset of choice sets under both designs. For a given choice set, regardless of whether it comes from the labeled or the unlabeled design, we can present it using either the labeled presentation style, where the mode names are displayed in the header row, or the unlabeled presentation style, where the mode names are displayed along with the other attributes. In this way, we obtain four choice task pools, each of which contains one type of questions to be answered by the respondents. For ease of exposition, let $P_{i}$ be the pool of choice tasks for question type *i* ∈ {1, 2, ⋯, 4}.

### Survey administration

Our goal is to assess the impact of the different question types to respondents’ choice behavior. According to [17], there are two approaches to administer the survey: 1) the split sample approach, where statistically equivalent samples are presented with different versions of the questionnaires; and 2) administer versions of the questionnaire in the same group of respondents where the order is completely randomized, as long as the the workload is appropriate. In our research, we adopt the second approach where the sample is presented with choice tasks from all four types of questions.

The survey is conducted on computers. When the respondent starts the survey, our computer program randomly selects three choice tasks from each of the four pools of choice tasks, that is, each of $P_{1}$, $P_{2}$, $P_{3}$, and $P_{4}$. These twelve choice tasks are then combined with a specially designed screening choice task which contains a dominant alternative (that is, an alternative that is logically preferable than the other alternatives in the choice set). This screening choice task is included in all questionnaires and is used to identify respondents with possible violations of rational preference. All thirteen questions are mixed in a random order to eliminate possible ordering effects among the choice tasks.

The pilot survey was administered in December 2012 to detect potential issues in the questionnaire. For example, we got inquiries from the respondents for Types 3 and 4 choice tasks. These two types of questions are generated by treating mode names as attributes in the design phase. As a result, the same mode may appear more than once in the choice tasks. Respondents asked us if we had made errors in the questionnaire. To dispel such confusion in the main survey, we explicitly told the respondents that the same mode might appear more than once and they needed to compare the detailed attribute levels to make choices. The main survey was conducted in May 2013. Both the pilot and the main surveys were administered in the city of Beijing by intercepting residents on the street. Since the concept of vehicle sharing is new to most Chinese residents, an introduction to vehicle sharing was given to the respondents before the survey. The introduction explained what vehicle sharing is, how to use shared vehicles, and the differences between vehicle sharing and traditional car rental. After they responded to all choice tasks, we also collected respondents’ socio-demographic information, such as their ages, whether they own private cars, and so on. We also solicited their responses to know more about their life styles. For example, we asked about their frequently used travel modes and their environmental attitude. This information can be used to identify the subpopulation that would be interested in vehicle sharing. In the main survey, we collected responses from a total of 230 individuals, among which 198 correctly answered the screening choice task.

## Results

As is said earlier, in our survey each respondent faces three choice tasks from each of the four question types. We group all responses for type *i* questions into data set *D*_*i*_, where *i* ∈ {1, 2, 3, 4}. We first investigate if the parameter estimates from the four data sets are the same in Section 5.1. We then estimate the conditional WTP for each individual and each question type, and use a random-effects model to relate its variation to the socioeconomic characteristics of the individuals as well as the treatment of alternatives’ names in Section 5.2.

### Impact toward parameter estimates

We first investigate if there are significant differences in the parameter estimates obtained from these four data sets. In the specification of the mixed logit models, we consider the following independent variables: the negative of in-vehicle time, the negative of out-of-vehicle time, the negative of the number of transfers, travel cost, parking cost, and three alternative-specific constants for private car (denoted as ASC_PC), vehicle sharing (denoted as ASC_VS), and taxi (denoted as ASC_TX). For the variables that we use the negatives, their respective coefficients are modeled using the log-normal distribution, because we want the utility to decline when the the values of those variables increase. The coefficients for the three alternative-specific constants, that is, ASC_PC, ASC_VS and ASC_TX, are assumed to be normally distributed, while those for travel cost and parking cost are assumed to be fixed numbers as is suggested by [33].

Let ***b***_*i*_ and ***W***_*i*_ be the coefficient parameters and *μ*_*i*_ be the scale parameter estimated using data set *D*_*i*_, where *i* = 1, 2, 3, 4. The estimation results are reported in Table 5. Columns 1 and 2 identify the coefficient parameters and the scale parameters. Columns 3 and 4 show the parameter estimates obtained from data set *D*_1_. The coefficient for transportation cost and parking cost are both negative, which is consistent with our expectation. In addition, the coefficient for parking cost is more negative in magnitude than that for travel cost, which implies that individuals are more sensitive to parking expenses. The means for the three alternative-specific constants are all negative, which indicates that travelers would prefer public transportation if all else are held equal. We also notice that the standard deviations of the coefficients are highly significant, which suggests that there is significant behavioral heterogeneity among the respondents. The value of the scale parameter, that is, *μ*_1_, is normalized to 1 for convenience. The results for data sets 2, 3, and 4 are shown in Columns 5 through 10, and similar observations can be made. Note that we shall not directly compare the parameter estimates reported in Columns 3 through 10, because their respective scale parameters are normalized to one.

**Table 5:** Estimation results for the mixed logit models.

Variables (1)	Parameters (2)	Data set *D*_1_		Data set *D*_2_		Data set *D*_3_		Data set *D*_4_		Pooled Data set 1 (Different scale parms.)		Pooled Data set 2 (Identical scale parms.)	
		Value (3)	S.E. (4)	Value (5)	S.E. (6)	Value (7)	S.E. (8)	Value (9)	S.E. (10)	Value (11)	S.E. (12)	Value (13)	S.E. (14)
In-vehicle time (neg.)	Mean of ln(coef.)	−3.0021^‡^	0.1781	−2.5938^‡^	0.1550	−3.2074^‡^	0.1450	−3.0022^‡^	0.1285	−2.9642^‡^	0.0715	−2.9350^‡^	0.0742
	S.D. of ln(coef.)	1.4942^‡^	0.4097	0.7348^‡^	0.2310	0.9461^‡^	0.2210	1.3528^‡^	0.3521	0.8904^‡^	0.1671	0.9605^‡^	0.1873
Out-of-vehicle time (neg.)	Mean of ln(coef.)	−3.2177^‡^	0.1887	−3.5634^‡^	0.4751	−3.5729^‡^	0.4381	−4.9587^‡^	0.6091	−3.4616^‡^	0.2083	−3.4650^‡^	0.2194
	S.D. of ln(coef.)	1.1561^‡^	0.3023	2.5835^†^	1.1595	0.9282^†^	0.4448	2.8959^†^	1.1505	1.1742^‡^	0.3731	1.2834^‡^	0.4087
No. of transfers (neg.) (Specific to public transport.)	Mean of ln(coef.)	−0.4098*	0.2456	−0.5850^†^	0.2904	−1.2246^†^	0.2606	−0.9408^‡^	0.2109	−1.7102^†^	0.4376	−1.8903^†^	0.5240
	S.D. of coef.	2.4049^†^	1.0679	2.7559^†^	1.1154	1.4520^†^	0.6075	1.5409^†^	0.6177	4.3402*	2.2466	5.0992^†^	2.3802
ASC_PC (Specific to private car)	Mean of coef.	−0.2109	0.2685	−0.7477*	0.4102	−0.4149	0.3709	−0.9621^‡^	0.3402	−0.6128^‡^	0.1831	−0.6292^‡^	0.1913
	S.D. of coef.	2.2965^‡^	0.7152	8.1717^‡^	2.9035	4.1294^†^	1.8717	2.2261^‡^	0.6829	4.8320^‡^	1.0753	5.7049^‡^	1.1437
ASC_VS (Specific to vehicle sharing)	Mean of coef.	−0.1156	0.2444	−0.3718	0.3793	−0.0366	0.2477	−0.9626^‡^	0.2650	−0.3267*	0.1687	−0.2983*	0.1729
	S.D. of coef.	2.0229^†^	0.9167	6.9754^‡^	1.7912	2.0107^†^	0.7921	2.4146^‡^	0.8403	3.4518^‡^	0.8295	3.8671^‡^	0.7787
ASC_TX (Specific to taxi)	Mean of coef.	−0.0913	0.2045	−0.2747	0.2997	−0.1560	0.2350	−0.6545^†^	0.2820	−0.2908^†^	0.1447	−0.2809^†^	0.1481
	S.D. of coef.	1.4496^†^	0.5936	2.4592^‡^	-0.6545	1.2929*	0.6739	4.5363^‡^	1.4034	1.9860^‡^	0.5556	2.1792^‡^	0.5592
Travel cost	Coef.	−0.0630^‡^	0.0059	−0.0658^‡^	0.0066	−0.0530^‡^	0.0065	−0.0315^‡^	0.0073	−0.0477^‡^	0.0034	−0.0510^‡^	0.0036
Parking cost	Coef.	−0.0843^‡^	0.0127	−0.1009^‡^	0.0161	−0.0968^‡^	0.0133	−0.0741^‡^	0.0128	−0.0774^‡^	0.0058	−0.0827^‡^	0.0061
*μ*_1_		1.00								1.00		1.00	
*μ*_2_				1.00						1.04		1.00	
*μ*_3_						1.00				0.76		1.00	
*μ*_4_								1.00		0.74		1.00	
No. of observations		690		690		690		690		2760		2760	
Loglikelihood		-801.3		-790.5		-714.3		-707.5		-3028.4		-3034.6	
Adjusted *ρ*^2^		0.1476		0.1590		0.2386		0.2458		0.2041		0.2032	

^‡^ significant at 1% level

^†^ significant at 5% level

* significant at 10% level

Following the steps in Section 3.2, we first test if the coefficient parameters are the same across all these four data sets, that is, we test the following hypothesis:

$$\begin{array}{c} H_{1a:}\;b_{1}=b_{2}=b_{3}=b_{4}\;\text{and}\;W_{1}=W_{2}=W_{3}=W_{4}. \end{array}$$

To test *H*_1*a*_, we estimate an unrestricted model which contains four mixed logit models for *D*_1_, *D*_2_, *D*_3_, and *D*_4_, respectively. The estimation results have been reported in Columns 3 through 10 in Table 5 and the corresponding loglikelihood for the unrestricted model is (−801.3) + (−790.5) + (−714.3) + (−707.5) = 3013.6. The restricted model is obtained by estimating a mixed logit model over the data matrix formed by pooling *D*_1_, *D*_2_, *D*_3_, and *D*_4_ together while allowing the scale parameters to be different. This is achieved by normalizing *μ*_1_ to 1 and systematically varying the values of *μ*_2_, *μ*_3_, and *μ*_4_ independently between 0 and 10.0 with a step size of 0.02. For each combination of *μ*_2_, *μ*_3_, and *μ*_4_, we can obtain the maximum value for the loglikelihood function. We find that the loglikelihood function reaches its maximum of -3028.4 when *μ*_2_ = 1.06, *μ*_3_ = 0.76, and *μ*_4_ = 0.72. The corresponding parameter estimates are reported in Columns 11 and 12 of Table 5. The statistic for the likelihood ratio test is -2[-3028.4 - (-3013.6)] = 29.6, which is less than the critical value of the Chi-squared distribution with 39 degrees of freedom at the 5% level of significance ($\chi_{39,0.05}^{2}=54.57$). Therefore, we cannot reject *H*_1*a*_, which means that the coefficient parameters are identical across these four data sets.

Since *H*_1*a*_ stands, we proceed to check if the scale parameters are the same among these four data sets:

$$\begin{array}{c} H_{1b:}\;\mu_{1}=\mu_{2}=\mu_{3}=\mu_{4}. \end{array}$$

This is accomplished by estimating the unrestricted model reported in Columns 11 and 12 in Table 5 and a restricted model over the data matrix formed by pooling all four data matrices together while restricting their scale parameters to be identical. The parameter estimates for the restricted model are reported in Columns 13 and 14 in Table 5. Note that the values of the scale parameters are all normalized to 1.0 and the loglikelihood is -3034.6. The test statistic for *H*_1*b*_ is -2[-3034.6-(-3028.4)] = 12.8, which exceeds the critical value of the Chi-square distribution with 3 degrees of freedom at the 5% level of significance ($\chi_{3,0.05}^{2}=7.82$). Hence, the hypothesis is rejected and there exits significant differences in the scale parameters of the four data sets. The findings related to *H*_1*a*_ and *H*_1*b*_ can be summarised as follows:

**Finding 1** *The labeled or the unlabeled treatment of alternatives’ names in either the design or the presentation phases of the choice experiment does not statistically affect the estimates of the coefficient parameters*. *However*, *these different treatments do cause the scale parameters to differ*.

**Fig 2:**
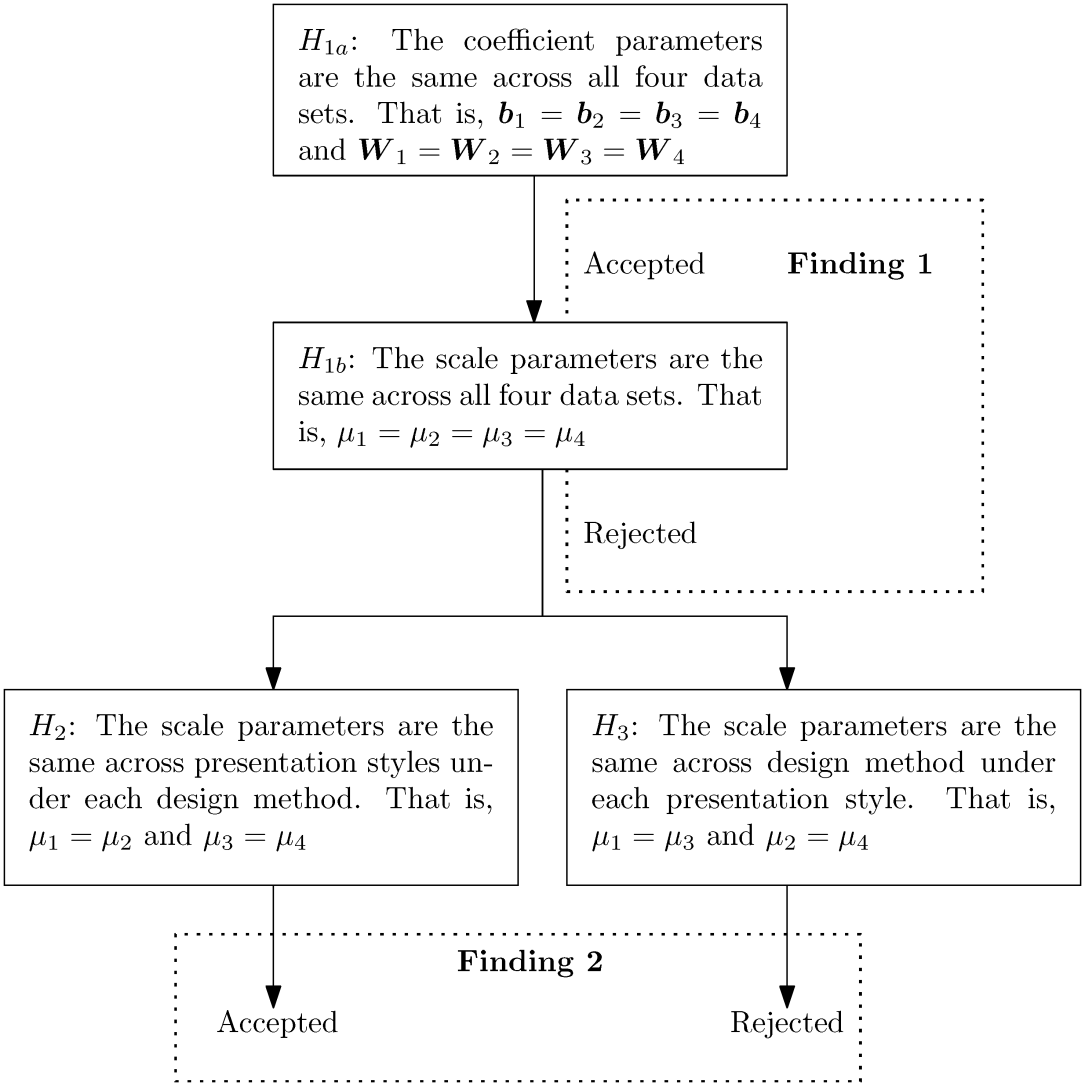
The hypothesis tests we conduct on the four data sets. The results are summarized into Findings 1 and 2 in the text.

The sequence of the above hypothesis tests is illustrated by the flow chart shown in Fig 2. At the top of the figure, we test *H*_1*a*_ and accept this hypothesis. Afterwards, we test *H*_1*b*_ and it gets rejected. These two results are summarized into Finding 1. Since *H*_1*b*_ is rejected, which means that the scale parameters are not identical across the four data sets, we conduct additional hypothesis tests to further analyze the cause: Is it due to the treatment of alternatives’ names in the design or in the presentation phase? This is accomplished by testing *H*_2_ and *H*_3_. Hypothesis *H*_2_ tests the following hypothesis: Given the treatment of alternatives’ names in the design phase, the scale parameters are the same between the labeled and the unlabeled treatments of alternatives’ names during the presentation phase. Mathematically, this means

$$\begin{array}{c} H_{2}:\;\mu_{1}=\mu_{2}\;\text{and}\;\mu_{3}=\mu_{4}. \end{array}$$

Hypothesis *H*_3_ tests the following hypothesis: Given the treatment of alternatives’ names in the presentation phase, the scale parameters are the same between the labeled and the unlabeled treatments of alternatives’ names during the design phase. That is,

$$\begin{array}{c} H_{3}:\;\mu_{1}=\mu_{3}\;\text{and}\;\mu_{2}=\mu_{4}. \end{array}$$

The details for the likelihood ratio tests associated with *H*_1*a*_, *H*_1*b*_, *H*_2_, and *H*_3_ are conveniently summarized in Table 6. Column 1 shows the hypothesis in question. Columns 2 and 3 show the data matrices and the loglikelihood function value for the unrestricted model. For example, while testing *H*_1*a*_ in Row 1, the log likelihood based on data matrices *D*_1_, *D*_2_, *D*_3_, and *D*_4_ are -801.3, -790.5, -714.3, and -707.5, respectively. Columns 4 and 5 show the data matrix for the restricted model and the log likelihood function value. The values of the scale parameters are also reported if applicable. In the test for *H*_1*a*_, the loglikelihood function of the restricted model reaches its maximum value of -3028.4 when *μ*_1_ = 1, *μ*_2_ = 1.06, *μ*_3_ = 0.76, and *μ*_4_ = 0.72. Finally, Column 6 shows the details of the likelihood ratio test, including the test statistic, the degree of freedom and critical value for the *χ*^2^ test, and whether the hypothesis is accepted or rejected. Rows 2 and 3 show the results for *H*_1*a*_ and *H*_1*b*_, respectively. In Row 4, we show that *H*_2_ is accepted, meaning that the treatment of alternatives’ names in the presentation phase does not affect the magnitude of the scale parameter. In Row 5, we show that *H*_3_ is rejected, meaning that the treatment of alternatives’ names in the design phase does affect the magnitude of the scale parameter.

**Table 6:** Detailed steps for the likelihood ratio tests.

Hypothesis		Unrestricted model		Restricted model		Result
		Data matrix	Log(L)	Data matrix	Log(L)	
*H*_1*a*_	*b*_1_ = *b*_2_ = *b*_3_ = *b*_4_; ***W***_1_ = ***W***_2_ = ***W***_3_ = ***W***_4_	*D*_1_	-801.3	$\left[\begin{array}{c} D_{1}\\ \mu_{2}D_{2}\\ \mu_{3}D_{3}\\ \mu_{4}D_{4} \end{array}\right]$	-3028.4 *μ*_2_ = 1.04 *μ*_3_ = 0.76 *μ*_4_ = 0.74	Test statistic = 29.6 Degree of freedom = 39 Critical value = 54.57 Hypothesis is accepted
		*D*_2_	-790.5			
		*D*_3_	-714.3			
		*D*_4_	-707.5			
*H*_1*b*_	*μ*_1_ = *μ*_2_ = *μ*_3_ = *μ*_4_	$\left[\begin{array}{c} D_{1}\\ \mu_{2}D_{2}\\ \mu_{3}D_{3}\\ \mu_{4}D_{4} \end{array}\right]$	-3028.4 *μ*_2_ = 1.04 *μ*_3_ = 0.76 *μ*_4_ = 0.74	$\left[\begin{array}{c} D_{1}\\ D_{2}\\ D_{3}\\ D_{4} \end{array}\right]$	-3034.6	Test statistic = 12.4 Degree of freedom = 3 Critical value = 7.82 Hypothesis is rejected
*H*_2_	*μ*_1_ = *μ*_2_ and *μ*_3_ = *μ*_4_	$\left[\begin{array}{c} D_{1}\\ \mu_{2}D_{2}\\ \mu_{3}D_{3}\\ \mu_{4}D_{4} \end{array}\right]$	-3028.4 *μ*_2_ = 1.04 *μ*_3_ = 0.76 *μ*_4_ = 0.74	$\left[\begin{array}{c} D_{1}\\ D_{2}\\ \mu_{3}D_{3}\\ \mu_{3}D_{4} \end{array}\right]$	-3029.6 *μ*_3_ = 0.74	Test statistic = 2.4 Degree of freedom = 2 Critical value = 5.99 Hypothesis is accepted
*H*_3_	*μ*_1_ = *μ*_3_ and *μ*_2_ = *μ*_4_	$\left[\begin{array}{c} D_{1}\\ \mu_{2}D_{2}\\ \mu_{3}D_{3}\\ \mu_{4}D_{4} \end{array}\right]$	-3028.4 *μ*_2_ = 1.04 *μ*_3_ = 0.76 *μ*_4_ = 0.74	$\left[\begin{array}{c} D_{1}\\ \mu_{2}D_{2}\\ D_{3}\\ \mu_{2}D_{4} \end{array}\right]$	-3034.5 *μ*_2_ = 0.92	Test statistic = 12.2 Degree of freedom = 2 Critical value = 5.99 Hypothesis is rejected

Based on the above results, ***β***_*tn*_ can be assumed to come from the same distribution with parameters ***b*** and ***W***, for all *t*. As a result, the posterior distribution of ***b*** and ***W*** based on all four data sets is given by

$$\begin{array}{c} K\left(b,W,\beta_{tn}\;\forall n\;\forall t|Y\right)\propto \prod_{t=1}^{T}\prod_{n=1}^{N}L\left(y_{tn}|\beta_{tn}\right)\phi \left(\beta_{tn}|b,W\right)k\left(b,W\right), \end{array}$$ (10)

where ***Y*** = (***Y***_1_, ***Y***_2_, ***Y***_3_, ***Y***_4_). This posterior is used in the conditional WTP analysis because it utilizes all data sets in a single model.

### Impact toward willingness-to-pay measures

An important output of behavioral models is the WTP measures. In our mode choice study, we are particularly interested in the WTP estimate for vehicle sharing. On one hand, we want to know whether the WTP estimate is affected by the design types and presentation styles; on the other hand, we are also keen to identify the sub-population that would be interested in vehicle sharing, that is, the individuals that have higher WTP for vehicle sharing, which shall help vehicle sharing companies better target their customers.

The panel is created by pooling the WTP estimates for the four types of questions for each individual. We then relate these individual level WTP estimates to the socioeconomic characteristics of each individual as well as the treatment of alternatives’ names in the design and the presentation phases. For a choice task, let indicator variable LBD_DSGN take value 1 if it is produced by treating the alternatives’ names as labels in the design phase; and zero, otherwise. Similarly, let indicator variable LBD_PRSN take value 1 if this question is produced by treating the alternatives’ names as labels in the presentation phase; and zero, otherwise. For example, for a choice task that belongs to Type 1 question in Table 3, both LBD_DSGN and LBD_PRSN equal 1. To explain the variability in *WTP*_*tnj*_, we specify the following random-effects model:

$$\begin{array}{c} WTP_{tnj}=\gamma^{\prime }z_{n}+\delta LBD\_DSGN+\pi LBD\_PRSN+\left(\alpha +\nu_{n}\right)+\omega_{nt}, \end{array}$$

where *z*_*n*_ is a vector of individual *n*’s socioeconomic characteristics, and *γ* is the corresponding coefficient vector; *δ* and *π* are the coefficients for the two dummy variables; *α* is the intercept term and is constant for all individuals; *ν*_*n*_ is the random heterogeneity specific to individual *n* and is constant across question types faced by individual *n*; and *ω*_*nt*_ is the error term. The estimate for the coefficient vector *γ* can uncover the socioeconomic causes for the preference heterogeneity toward attribute *j*, while those for *δ* and *π* can quantify the impact of labeled versus unlabeled treatments of alternatives’ names. Note that since we have a separate individual error term, the above model allows us to include individual specific socioeconomic variables in the regressors.

Table 7 summarizes the dependent and independent variables used in the model. The dependent variable is the WTP estimates for vehicle sharing. The independent variables are classified into four groups, where the first group is related to the socio-demographic characteristics of the respondents: the age and gender of the respondent, and whether he/she owns a vehicle. The age of the respondent is classified into three dummy variables: AGE1 takes value 1 if the age of the respondent is no more than 22 years old, and zero otherwise; AGE2 takes value 1 if the age of the respondent is greater than 22 years old but no more than 27 years old; and AGE3 takes value 1 if the age of the respondent is greater than 27 years old, and zero otherwise. This type of demographic information can be easily obtained from public sources and the insights from the random-effects model can be used to identify the sub-population that would be interested in vehicle sharing. The second group is about the life styles of the respondents, that is, whether he or she is a frequent user of public transportation, private car, or taxi; and whether he/she agrees with the statement that “I consider the environmental impact of the transportation mode I use”. The third group of variables indicate the design type and presentation style of the questions: Dummy variables LBD_DSGN and LBD_PRSN distinguish the treatment of alternatives’ names in the design and the presentation phase, respectively. The last group of variables detects possible violations of rational preference. Dummy variable NON_MON takes value 1 if the respondent fails to select the dominant alternative in the specially designed screening choice set. Note that the violation of non-monotonic preference does not necessarily imply theoretical inconsistency or irrationality of the respondent [5]. It may be due to the fact that the respondents have perceptions of missing attributes. As a result, these responses are not excluded from the WTP estimation. However, it is expected that these anomalous individuals shall have greater WTP estimates.

**Table 7:** Variables used in the random-effects model to explain the heterogeneity in the WTP estimate for vehicle sharing.

Variable	Description
***Dependent variable***	
WTP_VS	Conditional WTP for vehicle sharing (specific to each individual and question type)
***Socio-demographic characteristics***	
AGE1	Dummy variable that equals 1 when the respondent is no more than 22 years old; and zero, otherwise
AGE2	Dummy variable that equals 1 when the respondent is greater than 22 years old but no more than 27 years old; and zero, otherwise
AGE3	Dummy variable that equals 1 when the respondent is greater than 27 years old; and zero, otherwise
OWN_CAR	Dummy variable that equals 1 when the respondent owns a private car; and zero, otherwise
***Life Style***	
FRQT_PT	Dummy variable that equals 1 when the respondent is a frequent user of public transportation; and zero, otherwise
FRQT_TX	Dummy variable that equals 1 when the respondent is a frequent user of taxi; and zero, otherwise
ENV_FRNDLY	Dummy variable that equals 1 when the respondent reports that he/she considers the environmental impact of the travel mode he/she uses; and zero, otherwise
***Treatment of alternatives’ names***	
LBD_DSGN	Dummy variable that equals 1 when mode names are treated as labels in the design phase; and zero, otherwise
LBD_PRSN	Dummy variable that equals 1 when mode names are presented as labels in the presentation phase; and zero, otherwise
***Possible violation of rational preference***	
NON_MON	Dummy variable for non-monotonic preferences. It equals 1 when the respondent failed to choose the dominant alternative in the specially designed screening choice taskp

The estimation results for the random-effects model are reported in Table 8. We find that the coefficients for the age dummy variables, that is, AGE1 and AGE3, are not significant, which suggests that the interest in vehicle sharing does not vary significantly across different age groups. This finding is similar to that reported in [44], where the authors find that the coefficient for age is not significant. The coefficient for OWN_CAR is positive and significant, which means that vehicle owners are highly interested in vehicle sharing. This is in line with existing findings that vehicle sharing programs are fairly attractive to vehicle owners and contribute to the reduction in vehicle ownership [41]. For the three life style related variables, the coefficient for FRQT_PT is negative and significant, implying that public transportation users are not quite interested in vehicle sharing. This is understandable because the cost of vehicle sharing is much higher than that for public transportation. The coefficient for FRQT_TX is positive and significant, implying that frequent users of taxis have a higher WTP for vehicle sharing. This means that vehicle sharing service providers can launch targeted marketing campaigns toward taxi passengers at taxi hailing stations or through smartphone-based taxi hailing applications. The coefficient for ENV_FRNDLY is positive but insignificant, which suggests that the interest in vehicle sharing is only weakly correlated with the environment attitude of the individuals. The above results can be summarized into the following finding:

**Finding 2** *We find that individuals who are vehicle owners and who frequently use taxis have higher WTP for vehicle sharing*. *However*, *it appears that age and environmental attitude do not affect the WTP estimate*.

**Table 8:** Parameter estimates for the random-effects model.

	Coefficient	S.E.
Intercept	-10.2720^‡^	3.2507
AGE1	-1.1083	2.1138
AGE3	3.2682	2.3419
OWN_CAR	4.7624^†^	1.8839
FRQT_PT	-6.0769^‡^	2.2323
FRQT_TX	1.5060^†^	1.8702
ENV_FRNDLY	1.2559	1.9240
LBD_DSGN	0.9204	1.0859
LBD_PRSN	2.3303^†^	1.0859
NON_MON	13.5620^‡^	2.5832
S.D. of *ν*_*n*_	10.0510	
S.D. of *ω*_*nt*_	16.4690	
Lagrange multiplier test	76.7200	
Adjusted *R*^2^	0.3045	
Num. of observations	920	

^‡^ significant at 1% level

^†^ significant at 5% level

The coefficient for LBD_DSGN is not significant, which suggests that there is not much difference in the WTP estimates between the labeled and unlabeled treatments of alternatives’ names in the design phase. However, the coefficient for LBD_PRSN is positive and significant at the 5% level of significance, implying that the labeled treatment of alternatives’ names leads to higher WTP estimate than the unlabeled treatment in the presentation phase. This is consistent with our expectation, because when the names of the modes are displayed prominently in the header row, they are likely to become the target of choice and the actual attributes of the alternatives may be overlooked by the respondents, which would cause the WTP estimate to be greater. The dummy variable NON_MON is used to test the sensitivity of WTP for respondents who failed to detect dominant alternatives in the screening choice task. In total, 32, or 14%, respondents did not recognize the dominant alternative. The coefficient for this variable is positive and significant at the 1% level of significance, which is consistent with existing studies that respondents who fail to detect the dominant alternative tend to have higher WTP estimates than those who do [5]. The above results are summarized into the following finding:

**Finding 3** *Given the treatment of alternatives’ names in the presentation phase*, *the treatment of alternatives’ names in the design phase does not statistically affect the estimates of the WTP measures*. *However*, *given the treatment of alternatives’ names in the design phase*, *the labeled treatment of alternatives’ names in the presentation phase causes the corresponding WTP estimates to be slightly higher*.

Now let us look at the standard deviations of the two error terms. We notice that the standard deviation of *ν*_*n*_ is comparable to that of *ω*_*nt*_, which suggests that there is indeed considerable individual specific variability in the responses. To formally evaluate the validity of the random-effects model, the Lagrange multiplier test, developed by [47], is conducted to test the null hypothesis if the variance of *ν*_*n*_ is zero. The test statistic is 76.7200, which exceeds the critical value of the Chi-square distribution at the 5% level of significance ($\chi_{1,0.05}^{2}=3.84$). Hence, we can reject the null hypothesis and conclude that the random-effects model is more appropriate than ordinary least squares, implying there are significant differences across individuals.

## Conclusions

Discrete choice experiments have been widely applied to elicit behavioral preference in the literature. In many of these experiments, the alternatives are *named alternatives*, meaning that they are naturally associated with specific names. A fundamental issue that arises in stated choice studies is whether to adopt the labeled or unlabeled treatment of alternatives’ names in the design as well as the presentation phases of the choice sets. In the design phase, the labeled treatment of alternatives’ names views the alternatives’ names as labels and use the labeled design methods to generate the choice tasks, while the unlabeled treatment views the alternatives’ names as attributes and use the unlabeled design methods instead. In the presentation phase, the labeled treatment of alternatives’ names displays the alternatives’ names in the header row of the choice task, while the unlabeled treatment displays the alternatives’ names together with its attributes and use generic names in the header row to reference the alternatives.

We investigate the impact of labeled versus unlabeled treatments of alternatives’ names on the outcome of stated choice experiments. In a study that aims at eliciting Chinese residents’ preferences toward vehicle sharing, we generate four types of survey questions that differ by the treatment of alternatives’ names in the design and presentation phases. We estimate mixed logit models based on the collected data and find that parameter estimates are not affected by the treatment of alternative’s names at the design as well as the presentation phases. However, we do observe considerable differences in the scale parameters, which is attributable to the treatment of alternatives’ names in the design phase. We proceed to quantify the differences in WTP estimates. By building a random-effects model after we obtain the conditional WTP estimates for each individual and each question type, we find that the treatment of alternatives’ names during the design phase does not affect the WTP estimates, while that during the presentation phase does. Specifically, we find that the labeled treatment of alternatives’ names in the presentation phases produces slightly greater WTP estimates. We also find that vehicle owners and frequent users of taxis have higher WTP for vehicle sharing.

## Supporting information

S1 File The data set used in this study. (ZIP)
